# Mitochondrial dysfunction associated with glucocerebrosidase deficiency

**DOI:** 10.1016/j.nbd.2015.09.006

**Published:** 2016-06

**Authors:** Matthew E. Gegg, Anthony H.V. Schapira

**Affiliations:** Department of Clinical Neuroscience, UCL Institute of Neurology, London NW3 2PF, UK

**Keywords:** Autophagy, Gaucher disease, Glucocerebrosidase, Lysosome, Mitochondria, mitophagy, Parkinson's disease

## Abstract

The lysosomal hydrolase glucocerebrosidase (GCase) is encoded for by the *GBA* gene. Homozygous *GBA* mutations cause Gaucher disease (GD), a lysosomal storage disorder. Furthermore, homozygous and heterozygous *GBA* mutations are numerically the greatest genetic risk factor for developing Parkinson's disease (PD), the second most common neurodegenerative disorder. The loss of GCase activity results in impairment of the autophagy‐lysosome pathway (ALP), which is required for the degradation of macromolecules and damaged organelles. Aberrant protein handling of α-synuclein by the ALP occurs in both GD and PD. α-synuclein is the principle component of Lewy bodies, a defining hallmark of PD. Mitochondrial dysfunction is also observed in both GD and PD. In this review we will describe how mitochondria are affected following loss of GCase activity. The pathogenic mechanisms leading to mitochondria dysfunction will also be discussed, focusing on the likely inhibition of the degradation of mitochondria by the ALP, also termed mitophagy. Other pathogenic cellular processes associated with *GBA* mutations that might contribute, such as the unfolding of GCase in the endoplasmic reticulum, calcium dysregulation and neuroinflammation will also be described. Impairment of the ALP and mitochondria dysfunction are common pathogenic themes between GD and PD and probably explain why *GBA* mutations increase the risk of developing PD that is very similar to sporadic forms of the disease.

## 1 Glucocerebrosidase

Glucocerebrosidase (GCase; also known as glucosylceramidase; EC 3.2.1.45) is a lysosomal enzyme involved in sphingolipid metabolism. GCase catabolises the substrate glucosylceramide (GlcCer) to glucose and ceramide. Ceramide is the hydrophobic membrane anchor of all sphingolipids and is recycled to generate new glycosphingolipids (e.g. gangliosides such as GM1, GM2, sulfatide) and sphingomyelins (van Echten-Deckert and Herget, 2006).

GCase is encoded by the *GBA* gene located on chromosome 1q21 and is comprised of 11 exons, encoding for a protein of approximately 62 kDa. Upon translation, GCase is transported to the lysosome by binding to the transport receptor LIMP-2 in the endoplasmic reticulum (Reczek et al., 2007).

### 1.1 Gaucher disease

Autosomal recessive *GBA* mutations (homozygous or compound heterozygote) cause Gaucher disease (GD), the most common lysosomal storage disorder. Over 300 *GBA* mutations have been identified to cause GD (Hruska et al., 2008, Sidransky et al., 2009). Mutations lead to loss of GCase activity in the lysosome, resulting in the accumulation of GlcCer (Nilsson and Svennerholm, 1982, Nilsson et al., 1985). Accumulation of substrate in the lysosomes of macrophages is the main manifestation in visceral organs, leading to hepatosplenomegaly, anaemia, thrombocytopenia and bone involvement (Grabowski, 2008). While the above manifestations are common to all GD patients, GD is further classified in to non-neuronopathic (Type 1; OMIM#2308000) and neuronopathic (types 2 and 3; OMIM#23099 and OMIM 2301000, respectively). Onset of type 1 occurs in childhood or adulthood. Type 2 is the most severe, with substantial neurodegeneration and a median age of death at 9 months. Type 3 also has neurodegeneration, with death in childhood or early adulthood (Grabowski, 2008).

*GBA* mutations include point mutations, insertions, deletions, frame shifts, splice-site alterations and recombinant alleles, with patients showing considerable clinical heterogeneity despite similar genotypes. However, the mutant *N370S* allele, even in combination with another *GBA* mutant allele is predictive of Type 1 GD (Grabowski, 2008, Sun et al., 2013). Furthermore the *L444P* allele is strongly associated with neuronopathic GD, with a combination of *L444P* and another complex allele leading to type 2, and homozygous *L444P* mutations resulting in type 3. The *N370S* and *L444P* allele are the most common mutations associated with GD (Hruska et al., 2008).

Studies have shown that the intrinsic catalytic activity of N370S and L444P mutant GCase to be reduced by 80–95% compared to wild-type (Grace et al., 1999, Liou et al., 2006, Salvioli et al., 2005). Biochemical and molecular dynamic studies have suggested that N370S GCase is less able to associate with the physiological GCase activator saposin C and anionic phospholipids (Offman et al., 2010, Salvioli et al., 2005). However, loss of GCase activity is not solely due to impaired catalytic activity, but also a reduction in GCase protein levels. Many GCase mutations, including N370S and L444P, unfold in the endoplasmic reticulum (ER) at neutral pH, whereupon they are extracted by chaperones and degraded by the proteasome (Mu et al., 2008, Ron and Horowitz, 2005). This process is known as ER-associated degradation (ERAD).

### 1.2 Parkinson's disease

Parkinson's disease (PD) is the second most common neurodegenerative disorder. The loss of dopaminergic neurons in the substantia nigra results in movement disorders such as resting tremor, bradykinesia, rigidity and postural instability (Schapira and Jenner, 2011). In addition to neurodegeneration, PD is characterised by the presence of Lewy bodies in surviving neurons, intracellular protein inclusions predominantly consisting of α-synuclein. Mitochondrial dysfunction is also associated with PD pathogenesis (reviewed by Schapira and Gegg, 2011). Decreased activity of complex I of the electron transport chain (ETC) occurs in the substantia nigra of PD brains (Schapira et al., 1989). Several genes associated with early-onset autosomal recessive PD such as DJ-1, parkin and PINK1 have further strengthened the link between mitochondria and PD (Bonifati et al., 2003, Kitada et al., 1998, Valente et al., 2004). In particular, PINK1, a mitochondrial serine threonine kinase, and parkin, an E3 ubiquitin ligase, have recently been identified as key players in the identification and removal of damaged mitochondria by macroautophagy (Matsuda et al., 2010, Narendra et al., 2008, Vives-Bauza et al., 2010). The role of impaired autophagy in both PD and GD will be discussed further below.

### 1.3 *GBA* mutations and PD

In 1996 it was first reported that some type 1 GD patients exhibited typical Parkinsonism (Neudorfer et al., 1996). Further investigations then indicated that people with heterozygote *GBA* mutations were at greater risk of developing PD. A multicenter meta-analysis containing over 5000 PD patients reported the odds ratio of a PD patient carrying a *GBA* mutation to be 5.4 (Sidransky et al., 2009). Approximately 5–10% of PD patients carry a *GBA* mutation, making these mutations the most numerical genetic risk factor for developing PD. See Table 1 for a table summarising the main studies linking *GBA* with PD.

The age of onset of PD in patients with *GBA* mutations is approximately 5 years earlier than sporadic PD cases (Neumann et al., 2009, Sidransky et al., 2009). GBA-associated parkinsonism resembles sporadic PD, with no noticeable changes in Lewy body pathology reported either (Neumann et al., 2009, Parkkinen et al., 2011). However, cognitive impairment is more frequent in PD patients with GBA mutations (Alcalay et al., 2012, Beavan et al., 2015, Brockmann et al., 2011). It should be noted that *GBA* mutations are also significantly associated with developing dementia with Lewy bodies (DLB) and PD with dementia (odds ratios of 8.3 and 6.5, respectively; Nalls et al., 2013).

The two most frequent GBA mutations associated with PD are N370S and L444P, accounting for up to 17–31% of all PD patients in the European Ashkenazi Jewish population, and 3% in non-Ashkenazi populations (Neumann et al., 2009, Sidransky et al., 2009). Biochemical analysis of PD brains with *GBA* mutations indicated a significant decrease in GCase activity, with the greatest deficiency (58% decrease in enzyme activity) occurring in the substantia nigra (Gegg et al., 2012). Western blot analysis indicated that this loss of activity was in part due to a decrease in protein expression. Markers of the unfolded protein response (UPR) were increased in PD brains with GBA mutations, suggesting that mutant GCase is degraded by ERAD (Gegg et al., 2012).

Notably, GCase activity and protein expression was also significantly decreased by 33% in the substantia nigra of sporadic PD brains (Gegg et al., 2012). Another study indicated that GCase protein expression was significantly decreased in the anterior cingulate cortex of sporadic PD brains (Murphy et al., 2014). This study also showed that the decreased GCase protein levels in the brain significantly correlated with increased α-synuclein levels. This supports cell culture studies that had indicated that increased α-synuclein levels decreased GCase protein levels by inhibiting the trafficking of GCase to the lysosome (Gegg et al., 2012, Mazzulli et al., 2011).

### 1.4 Lysosomal dysfunction in GD and PD

The accumulation of GlcCer in lysosomes of GD patients and the well documented lysosomal dysfunction associated with PD (Alvarez-Erviti et al., 2010, Cuervo et al., 2004, Dehay et al., 2010), has meant that much of the GCase research in recent years has focused on the autophagy lysosome pathway (ALP). However it is important to note that the mechanisms leading to GD and GD patients developing PD are unlikely to be identical to PD patients with heterozygote *GBA* mutations. The greatest disparity is likely to be the accumulation of GlcCer and GlcSph in peripheral organs and brains of type 1, 2 and 3 GD patients (Nilsson and Svennerholm, 1982, Nilsson et al., 1985, Orvisky et al., 2002). Mouse models of neuronopathic GD models also have accumulation in the brain (Enquist et al., 2007, Farfel-Becker et al., 2014, Sun et al., 2013). However, no accumulation of GlcCer or GlcSph was detected in PD brains with heterozygote *GBA* mutations (Gegg et al., 2015), or mouse models lacking one of the *GBA* alleles (*GBA* +/−; Farfel-Becker et al., 2014, Sardi et al., 2011). Analysis of whole brain homogenates does not differentiate between neurons, glia and other cell types in the brain. A very modest accumulation of GlcCer has been reported in cortical mouse neurons with 50% decreased GCase activity following knock down (KD) with RNAi (Mazzulli et al., 2011)and in neurons differentiated from inducible pluripotent stem cells (iPSC) containing heterozygote *GBA* mutations (Schöndorf et al., 2014).

**Fig. 1:**
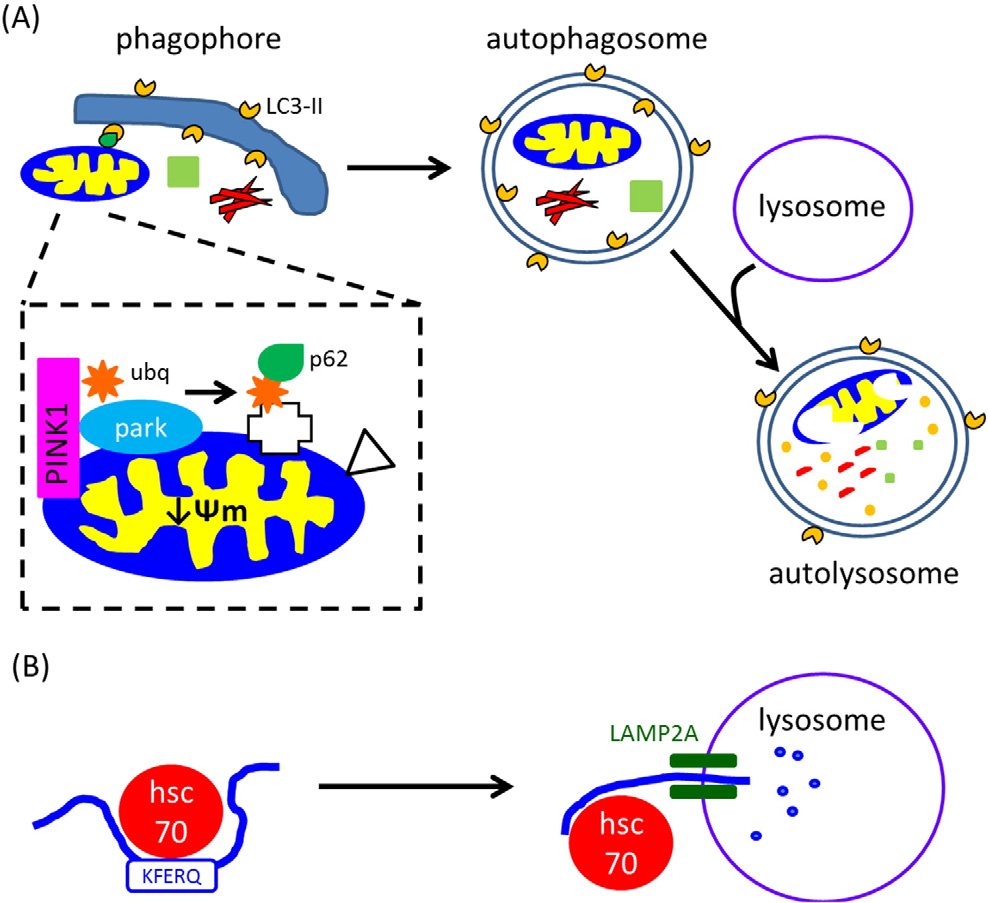
Macroautophagy and chaperone mediated autophagy pathways. (A) Macromolecules such as protein and lipids (green square), aggregated proteins (red fibrils) or damaged mitochondria (see inset) are recruited to phagophores by binding to LC3-II embedded in the membrane (orange segments). Phagophores mature into double membrane autophagosomes thus sequestering cargo for degradation. Following fusion with lysosomes to form an autolysosome, macromolecules and organelles are degraded by degradative enzymes from the lysosome. Inset: damaged mitochondria have decreased Ψm causing accumulation of full length PINK1 on the OMM. This recruits and phosphorylates the E3 ubiquitin ligase parkin (park) and ubiquitin (ubq; orange star), resulting in ubiquitination of proteins in the OMM (white cross). These proteins can then be bound by p62 (green teardrop) that enables binding to LC3-II on the phagophore. Other mitophagy receptors (white triangle) that can bind LC3-II such as FUNDC1, BNIP and cardiolipin are also up regulated following mitophagy induction. (B) Chaperone mediated autophagy degrades proteins with the pentapeptide motif KFREQ (approximately 30% of cellular proteins contain this motif). Unfolded protein is bound by hsc70, which then directly delivers protein to lysosomes for degradation via the integral protein LAMP2A.

While it is still unclear exactly how *GBA* mutations impact on lysosomal function, it is evident that loss of GCase activity effects the ALP in both heterozygote and homozygote *GBA* models. Two processes of the ALP are particularly relevant to GCase deficiency: chaperone mediated autophagy (CMA) and macroautophagy (Fig. 1). CMA involves the degradation of soluble proteins containing a KFERQ pentapeptide motif. Unfolded proteins are delivered to the lysosome via the chaperone hsc70, and the proteins are then directly translocated in to the lysosome for degradation via the integral membrane protein LAMP2A (reviewed by Mizushima et al., 2008, Parzych and Klionsky, 2014). Macroautophagy also degrades macromolecules such as protein and lipids, but also has the capacity to degrade larger structures, such as aggregated proteins and damaged organelles like mitochondria (Mizushima et al., 2008, Parzych and Klionsky, 2014). Cargo for degradation is engulfed by a phagophore membrane, which then expands to form a double-membrane cytosolic vesicle, known as an autophagsosome. The autophagosome then fuses with lysosomes, resulting in degradation of the inner membrane of the autophagosome and its sequestered cargo. Degradation of α-synuclein can occur via both CMA and macroautophagy.

Inhibition of GCase activity with the toxin conduritol β epoxide (CBE) resulted in the accumulation of α-synuclein in differentiated SH-SY5Y cells or in the brains of mice, just 48 h after administration (Manning-Boğ et al., 2009, Cleeter et al., 2013). The accumulation of α-synuclein has since been reported in primary neurons with *GBA* KD (50% loss of activity; Mazzulli et al., 2011), neurons differentiated from iPSC derived from GD patients and those carrying heterozygous *GBA* mutations (Mazzulli et al., 2011, Schöndorf et al., 2014) or in the brains of GD mouse models (Cullen et al., 2011, Mazzulli et al., 2011, Sardi et al., 2011, Osellame et al., 2013). The accumulation of α-synuclein in the brains of the D409V/D409V GD mouse model (e.g. hippocampus, cerebral cortex) were progressive, proteinase K-resistant and positive for ubiquitin, sharing characteristics with Lewy bodies found in PD (Sardi et al., 2011). Memory deficits were also associated with α-synuclein accumulation, and both could be ameliorated by injection of adeno-associated viral vectors encoding human GCase (Sardi et al., 2013, Sardi et al., 2011). Soluble and insoluble α-synuclein species have also been reported in another GD mouse model (V394L/V394L crossed on to the hypomorphic prosaposin mutant mice; Mazzulli et al., 2011). The authors also showed that GlcCer can also stabilise soluble oligomeric α-synuclein species in vitro (Mazzulli et al., 2011).

Primary neurons with approximately 50% GCase KD exhibited increased LC3-II levels, while GD neurons generated from iPSC had compromised proteolysis of long-lived proteins indicating that autophagy is affected by GCase deficiency (Mazzulli et al., 2011). The turnover of α-synuclein in L444P/wt mice carrying either human wild-type α-synuclein or human A53T α-synuclein was found to be decreased (Fishbein et al., 2014). Furthermore, the motor and gastro-intestinal deficits observed in the A53T mouse model were exacerbated when crossed with the L444P/wt mice (Fishbein et al., 2014). Inhibition of macroautophagy flux has been reported in primary neuronal cultures derived from GBA knock out (KO)mice (Osellame et al., 2013) and human neurons differentiated from iPSC derived from GD patients and people with heterozygote *GBA* mutations (Schöndorf et al., 2014). This inhibition was coincident with α-synuclein accumulation. The accumulation of α-synuclein in cell culture models over expressing mutant GCase could also be reversed by treatment with the macroautophagy activator rapamycin (Cullen et al., 2011). These data all indicate that the ALP is impaired with either heterozygote or homozygote *GBA* mutations.

Recent studies have strongly implicated the cell to cell transmission of misfolded proteins such as α-synuclein in the onset and progression of neurodegenerative disorders such as PD (Guo and Lee, 2014, Luk et al., 2012). Genetic ablation of *GBA* by zinc finger technology in SH-SY5Y cells increased cell to cell transmission of α-synuclein (Bae et al., 2014). Furthermore, when these cells were injected into the hippocampus of a transgenic mouse expressing human α-synuclein, the accumulation of host α-synuclein in the graft SH-SY5Y cells lacking *GBA* expression was greater than normal SH-SY5Y grafts (Bae et al., 2014).

### 1.5 Mitochondrial dysfunction and GBA

Loss of GCase activity in brain cells also results in loss of mitochondrial function. Inhibition of GCase by CBE in SH-SY5Y neuroblastoma cells resulted in a significant decrease in mitochondrial membrane potential (ψm) after 10 days, and decreased progressively up to 30 days of CBE treatment (Cleeter et al., 2013). Similarly, GCase KD with shRNA constructs decreased ψm (Cleeter et al., 2013). Generation of ψm by the mitochondrial ETC is used to drive the phosphorylation of ADP to form ATP. In CBE-treated cells, the phosphorylation of ADP was significantly inhibited after 20 days when glutamate and malate (complexes I, III, IV) or succinate (complexes II, III, IV) were used as substrates. However, ADP phosphorylation via complex IV only (ascorbate and TMPD as substrates) was unaffected (Cleeter et al., 2013). This inhibition of the ETC was coincident with an increase in oxidative stress and fragmentation of mitochondria.

Mitochondrial dysfunction has also been reported to occur in GD fibroblasts and GD mouse models. Dermal fibroblasts from three GD patients (L444P/L444P) exhibited impaired oxygen consumption when substrates for complex I, II, III and II + III of the ETC were used (de la Mata et al., 2015). Complex IV activity was not investigated. Notably, the levels of the respiratory chain electron carrier coenzyme Q_10_ (CoQ_10_) were reduced by 24% in these GD fibroblasts. ATP levels were decreased by 30% in these fibroblasts and there was decreased MitoTracker Red staining, suggestive of decreased ψm. Increased production of the free radical superoxide by mitochondria was also detected (de la Mata et al., 2015).

Single cell analysis of astrocytes and neurons derived from a type II GD KO mouse model indicated a loss of ψm (Osellame et al., 2013). Similar to the CBE model above, the activities of complex I, and II/III, but not complex IV, were inhibited in these mixed neuron–glia cultures (Osellame et al., 2013). Oxygen consumption (either basal or uncoupled) was consequently inhibited, and once again, mitochondria were also more fragmented in cells lacking GCase activity (Osellame et al., 2013).

The morphology of mitochondria in the cortices of a GD mouse model (D409H/D409H with hypomorphic prosaposin; 12 weeks old) was also affected as measured by electron microscopy (Xu et al., 2014). Mitochondria in these mice appeared to have lost cristae organisation, and were more rounded and electron dense. Steady-state ATP levels and basal mitochondria oxygen consumption were both decreased in the cortices of this mouse model, and four others (hypomorphic prosaposin, D409H/D409H, V394L/V394L, V394L/V394L with hypomorphic prosaposin). Inhibition of primary cortical neurons treated with CBE for 7 days also exhibited decreased oxygen consumption and lower steady-state ATP levels (Xu et al., 2014).

### 1.6 Pathogenic mechanisms for mitochondrial dysfunction following loss of GCase activity

**Fig. 2:**
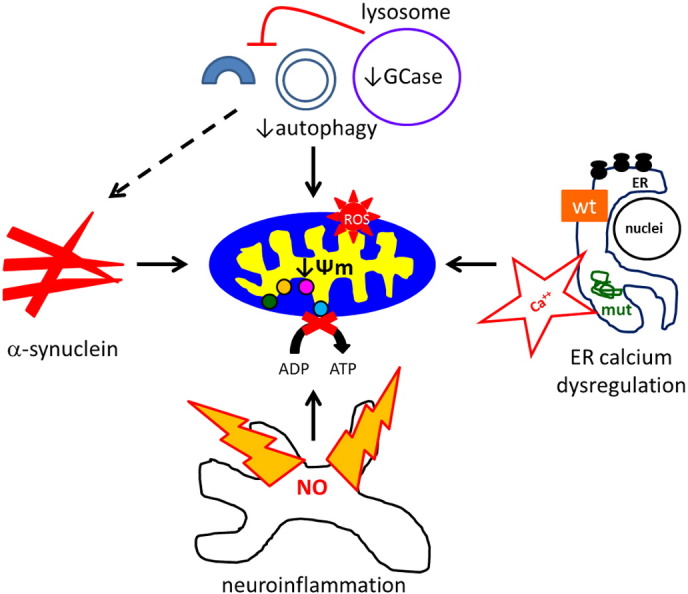
Putative pathogenic mechanisms for mitochondrial dysfunction following a loss of GCase activity. The mitochondrial dysfunction (loss of Ψm, ETC inhibition, reactive oxygen species (ROS)) observed following decreased GCase activity is most likely to be a result of inhibition of macroautophagy resulting in damaged mitochondria not being degraded by mitophagy. Inhibition of autophagic pathways also results in accumulation of α-synuclein, which can impair mitochondrial function. Dysregulation of calcium is another route by which mitochondrial function can be compromised. Increased calcium release from the ER is observed in *GBA* mutant cells. This in part is likely due to mutant GCase (mut) unfolding in the ER and activating ER stress. Neuroinflammation occurs in GD brains and mouse models. The production of NO by activated astrocytes and microglia will damage the mitochondrial ETC.

The loss of mitochondrial function in GCase deficient cells could be explained by impairment of the ALP, changes in cellular lipid/sterol metabolism, neuroinflammation, perturbed calcium homeostasis or a combination of the above (Fig. 2).

Damaged mitochondria are degraded by macroautophagy, and is also termed mitophagy (Kim et al., 2007, Tolkovsky et al., 2002). Mitochondria undergo fusion and fission forming a dynamic network within the cell. When mitochondria are damaged, they lose ψm, which results in fission from the network, whereupon they are recruited to a phagophore membrane for subsequent degradation (Twig et al., 2008; Fig. 1). Damaged mitochondria are selectively recruited to phagophores because they become decorated with several autophagic receptors that bind to LC3-II, which is embedded in the phagophore membrane. The mitochondrial serine/threonine kinase PINK1 and the E3 ubiquitin ligase parkin are two such proteins that are involved in identifying mitochondria that need to be degraded. Autosomal recessive mutations in the *PINK1* and *parkin* genes are a cause of early-onset familial PD (Kitada et al., 1998, Valente et al., 2004). Loss of ψm leads to the accumulation of full length PINK1 on the outer mitochondrial membrane (OMM), which then recruits parkin (Matsuda et al., 2010, Narendra et al., 2010, Vives-Bauza et al., 2010). PINK1-mediated phosphorylation of both parkin and ubiquitin (Kazlauskaite et al., 2014, Koyano et al., 2014) results in the ubiquitination of many OMM proteins, including VDAC1, mitofusins 1 and 2 (Gegg et al., 2010, Geisler et al., 2010, Sarraf et al., 2013). Some of these ubiquitinated proteins are then bound by the autophagic protein p62/SQSMT1, which then binds to LC3-II on the phagophore membrane (Ding et al., 2010, Lee et al., 2010). Other OMM proteins such as FUNDC1, BNIP3, Nix, and the mitochondrial lipid cardiolpin, have also been reported to bind LC3-II directly following mitophagy induction (Chu et al., 2013, Ding et al., 2010, Liu et al., 2012).

Impairment of mitophagy is likely to contribute to the mitochondrial dysfunction observed in PD and GD. The KD or KO of PINK1 leads to mitochondrial dysfunction and oxidative stress that worsens with time (Gautier et al., 2008, Gegg et al., 2009). Restoration of mitophagy by over expressing parkin in PINK1 KD cells reversed the inhibition of oxidative phosphorylation (Gegg et al., 2010). Mitochondrial dysfunction and oxidative stress following GCase inhibition by CBE in SH-SY5Y cells also increased over time, with no evidence of mitochondrial inhibition after 24 or 48 h, but increased after 10 days (Cleeter et al., 2013; Noelker et al., 2015). In the primary cultures from GBA KO mice and GD fibroblasts, the mitochondrial dysfunction observed was coincident with decreased macroautophagy flux (De la Mata et al., 2015, Osellame et al., 2013). Furthermore, the co-localisation of mitochondria and LC3 was decreased following induction of mitophagy in GBA KO cells (Osellame et al., 2013). These observations all imply that the degradation of mitochondria is decreased in GCase deficient cells, leading to the accumulation in cells of damaged mitochondria with impaired ETC producing damaging reactive oxygen species.

While inhibition of mitophagy is an attractive explanation for the mitochondrial dysfunction, it is perhaps not the full story. In theory, blockade of mitophagy should result in the impairment of all four ETC complexes. While there was a trend for complex IV to be decreased following CBE treatment of SH-SY5Y cells or in GBA KO brains, it was not significantly decreased like complexes I, II or III. Recently, endogenous CoQ_10_ levels have been reported to be decreased in GD fibroblasts, and treatment of cells with exogenous CoQ_10_ partially reversed the decrease in ATP levels and ψm (de la Mata et al., 2015). CoQ_10_ transfers electrons from both complexes I and II to complex III in the ETC (Hargreaves, 2014). The measurements of mitochondrial oxygen consumption and ADP phosphorylation described above rely on endogenous CoQ_10_, and therefore the reported decreases in mitochondrial function using substrates for complex I and II/III, could be a result of decreased CoQ_10_ levels. Indeed, when Cleeter et al. measured the individual activity of complex I by spectrophotometry in CBE-treated cells, which relies on the addition of exogenous CoQ, there was a trend for decreased complex I activity but it was no longer significant. However, the individual activity of complex I was still found to be significantly inhibited in GBA KO brain (Osellame et al., 2013). The reason for the decrease of CoQ_10_ in the GD fibroblasts is unclear and further reports are needed. Changes in lipid/sterol metabolism as a result of GCase inhibition may impact on the biosynthesis of CoQ_10_ (Hargreaves, 2014). Alternatively, CoQ10 is also known to play a role in the proton translocation across the lysosomal membrane (Matalonga et al., 2014), and the lysosomal accumulation of substrate in GD may have an effect on the lysosomal pool of CoQ_10_. Despite the uncertainty, it is becoming increasingly clear that treatment with CoQ_10_ or analogues such as MitoQ can partially reverse the mitochondrial dysfunction and oxidative stress observed in cell models of GD and other lysosomal storage disorders (De la Mata et al., 2015, Matalonga et al., 2014, Osellame et al., 2013).

The accumulation of α-synuclein in a variety of GCase deficient models described above may also have an impact on mitochondrial function. In neurons from the cortices of the D409H/D409H with hypomorphic prosaposin mice, α-synuclein was reported to accumulate in mitochondria (Xu et al., 2014). Mitochondria isolated from PD brains have been shown to have increased α-synuclein, with inhibition of complex I of the ETC correlating with the amount of α-synuclein in mitochondria (Devi et al., 2008). Soluble prefibrillar α-synuclein oligomers have also been observed to inhibit complex I, decrease ψm and disrupt calcium homeostasis (Luth et al., 2014). A similar scenario was reported in primary cultured neurons treated with synthetic preformed fibrils of α-synuclein (Dryanovski et al., 2013). After two weeks these exogenous fibrils had seeded α-synuclein aggregates of endogenous α-synuclein that resembled Lewy bodies and Lewy neurites. These neurons presented greater mitochondrial oxidant stress compared to their controls (Dryanovski et al., 2013).

Dysregulation of calcium can also have a profound effect on mitochondrial function, either by directly depolarising mitochondria (abolishing ψm) or oxidative damage following the generation of reactive oxygen species (Burchell et al., 2010). Calcium homeostasis is affected in both GD and heterozygous *GBA* models, with ER-buffering of calcium appearing to be most affected. Calcium release via the ER ryanodine receptor has been reported to be increased from rat brain microsomes treated with GlcCer or microsomes prepared from post-mortem type II GD brains (Lloyd-Evans et al., 2003, Pelled et al., 2005). Furthermore, basal cytosolic calcium levels are increased in neurons differentiated from L444P/L444P and L444P/wt iPSC (Schöndorf et al., 2014). Calcium release from the ER with the ryanodine receptor agonist caffeine was also increased in both these types of neurons. The expression of neuronal calcium binding protein 2 (NECAB2) was significantly increased in these neurons, and is likely to be a cytoprotective response, since NECAB2 KD in the GD and heterozygous *GBA* mutation neurons increased calcium-induced toxicity (Schöndorf et al., 2014). Since GCase mutations such as N370S and L444P are known to unfold in the ER, induce the UPR, and effect ER calcium homeostasis (Mu et al., 2008, Ron and Horowitz, 2005), it is likely that some of the changes in ER calcium handling observed in both GD and PD lines with heterozygous *GBA* mutations are related to this.

Finally, neuroinflammation may also impact on some of the mitochondrial abnormalities observed in GD models. The mitochondrial ETC is very susceptible to oxidative damage by reactive oxygen nitrogen species such as nitric oxide (NO) and peroxinitrite, a product of NO reacting with the free radical superoxide produced by the mitochondrial ETC (Bolaños et al., 1994, Bolaños et al., 1995, Cleeter et al., 1994). Activated astrocytes and microglia produce NO, and co-culturing of neurons with activated astrocytes results in inhibition of the neuronal mitochondrial ETC (Bolaños et al., 1996). Inhibition of the ETC was found to be reversible after 24 h of co-culture, but became irreversible after 48 h of co-culture (Stewart et al., 2000). Co-culturing of astrocytes with neurons approximately doubles the levels of the antioxidant glutathione in neurons, and this is due to the supply of glutathione precursors by astrocytes (Bolaños et al., 1996). This process limits the neuronal mitochondrial damage caused by astrocytic NO, as evidenced by the fact that mitochondrial damage was worse in neurons co-cultured with activated astrocytes that did not release glutathione precursors (Gegg et al., 2005).

Astrogliosis has been reported in type I (some with parkinsonism and dementia) or type II GD patients (Wong et al., 2004). The evidence of neuroinflammation is even greater in mouse GD models. In mice where *GBA* is KO in neurons and astrocytes only, activated astrocytes and microglia are an early event, and is closely coupled with neuronal loss and occurs prior to manifestation of disease (Enquist et al., 2007, Farfel-Becker et al., 2011). GlcCer accumulates in neurons throughout the brain prior to evidence of activated microglia. However, only certain regions of the brain exhibit neurodegeneration and activated microglia, indicating varying susceptibility of neuronal populations to GlcCer accumulation (Farfel-Becker et al., 2014). Activation of glia is also seen in mice treated with the GCase inhibitor CBE (Vitner et al., 2014), and V394L/V394L GD mice lacking prosaposin c (Sun et al., 2010).

## Abbreviations

ALP: autophagy lysosome pathway
AP: autophagosome
CBE: conduritol β epoxide
CoQ_10_: coenzyme Q_10_
DLB: dementia with Lewy bodies
ER: endoplasmic reticulum
ERAD: ER-associated degradation
ETC: electron transport chain
GCase: glucocerebrosidase
GD: Gaucher disease
GlcCer: glucosylceramide
iPSC: inducible pluripotent stem cells
KD: knock down
KO: knock out
NECAB2: neuronal calcium binding protein 2
NO: nitric oxide
OMM: outer mitochondrial membrane
PD: Parkinson's disease
UPR: unfolded protein response
ψm: mitochondrial membrane potential

## Conflict of interest

The authors do not have any conflicts of interest.

## Figures and Tables

**Table 1:** Summary of main studies linking GBA and PD.

Links between mutant GBA and PD	References
Subset of GD patients develop parkinsonism	Bembi et al. (2003), Neudorfer et al. (1996), Tayebi et al. (2001)
Genetic multi-centre study reporting statistically significant association between *GBA* mutations and PD	Sidransky et al. (2009)
Lewy bodies similar in PD brains with and without *GBA* mutations	Neumann et al. (2009), Parkkinen et al. (2011)
Accumulation and impaired turnover of α-synuclein in animal and cell models of GCase deficiency	Cullen et al. (2011), Fishbein et al. (2014), Manning-Boğ et al. (2009), Mazzulli et al. (2011), Osellame et al. (2013), Sardi et al. (2011), Schöndorf et al. (2014)
GCase activity decreased in sporadic PD	Gegg et al. (2012), Murphy et al. (2014)
α-synuclein affects GCase function	Gegg et al. (2012), Mazzulli et al. (2011), Sardi et al. (2013), Yap et al. (2011)
